# Multidrug-resistant Shigella flexneri outbreak associated with a high-mortality spillover event into nonhuman primates

**DOI:** 10.21203/rs.3.rs-4682172/v1

**Published:** 2024-07-12

**Authors:** Daryl Domman, Sarah Shrum Davis, Paris Salazar-Hamm, Karen Edge, Tim Hanosh, Jessica Houston, Anastacia Griego-Fisher, Francelli Lugo, Nicholas Wenzel, D’Eldra Malone, Carol Bradford, Kelly Plymesser, Michael Baker, Kurt Schwalm, Sarah Lathrop, Chad Smelser, Darrell Dinwiddie

**Affiliations:** University of New Mexico Health Sciences Center; University of New Mexico Emerging Infections Program; University of New Mexico; New Mexico Department of Health; New Mexico Department of Health; New Mexico Department of Health; New Mexico Department of Health; City of Albuquerque Environmental Health; New Mexico Department of Health; New Mexico Department of Health; City of Albuquerque BioPark; University of New Mexico Emerging Infections Program; University of New Mexico Emerging Infections Program; University of New Mexico Health Sciences Center; University of New Mexico Emerging Infections Program; New Mexico Department of Health; University of New Mexico Health Sciences Center

## Abstract

Shigellosis is a gastrointestinal infection caused by species of *Shigella*. A large outbreak of *Shigella flexneri* serotype 2a occurred in Albuquerque, New Mexico (NM) between May 2021 and November 2023 that involved humans and nonhuman primates (NHP) from a local zoo. We analyzed the genomes of 202 New Mexico isolates as well as 15 closely related isolates from other states, and four from NHP. The outbreak was initially detected within men who have sex with men (MSM) but then predominantly affected people experiencing homelessness (PEH). Nearly 70% of cases were hospitalized and there was one human death. The outbreak extended into Albuquerque’s BioPark Zoo, causing high morbidity and six deaths in NHPs. The NHP isolates were identical to those in the human outbreak. All isolates were multidrug-resistant, including towards fluoroquinolones, a first line treatment option which led to treatment failures in human and NHP populations. We demonstrate the transmission of this S. *flexneri* strain between humans and NHPs, causing fatalities in both populations. This study demonstrates the threat of antimicrobial resistant organisms to vulnerable human and primate populations and emphasizes the value of vigilant genomic surveillance within a One Health framework.

## Introduction

Shigellosis is a bacterial gastrointestinal infection caused by species of *Shigella* and characterized by bloody diarrhea, fever, and abdominal cramping, which may lead to extraintestinal complications such as sepsis, seizures, and reactive arthritis^1^. Shigellosis is caused by four species of *Shigella: S. sonnei, S. flexneri, S. dysenteriae*, and *S. boydii*, all of which are transmitted via the fecal-oral route. *Shigella* species have an low infectious dose of 10–100 cells^2^, and infection is common in young children, international travelers, men who have sex with men (MSM), and people experiencing homelessness (PEH)^1,3^. Within higher income settings such as Europe and the United States, many shigellosis cases have been attributed to transmission within the MSM community^4–6^. Additionally, there have been reports of recent outbreaks of shigellosis within PEH communities in several U.S. cities^4,7–9^.

While most shigellosis cases resolve naturally in short duration, antibiotic treatment may be recommended for severe cases and to limit the duration of infection and bacterial shedding^1^. The WHO first-line antimicrobial recommendation for shigellosis is ciprofloxacin, with pivmecillinam, ceftriaxone, or azithromycin as alternatives. The Infectious Diseases Society of America (IDSA) similarly recommends ciprofloxacin, azithromycin or ceftriaxone as first-line options, with trimethoprim-sulfamethoxazole or ampicillin as alternatives. Species of *Shigella* worldwide have acquired drug resistance to many first-line treatment options and genomic analyses have demonstrated the rapid emergence and spread of a fluoroquinolone resistant lineage for S. *sonnei*^1,10,11^. Many countries have recently reported *Shigella* strains designated extensively drug resistant (XDR), which display resistance to all empirical and alternative recommended antibiotics. In the U.S., prevalence of XDR *Shigella* rose from 0% in 2015 to 5% in 2022^3^.

Here, we present the epidemiological and genomic characterization of a large shigellosis outbreak first identified within the Albuquerque, NM metro area caused by a multidrug-resistant (MDR) S. *flexneri* strain. This outbreak affected both PEH and MSM populations, and is remarkable in that this outbreak strain spread to the nonhuman primate (NHP) population at a local zoo to caused significant morbidity and mortality within that population.

## Methods

### Epidemiologic information

We obtained shigellosis case data from the New Mexico Foodborne Diseases Active Surveillance Network (FoodNet), an active, population-based network which conducts surveillance for laboratory-confirmed infections, including species of *Shigella*. Our case definition was: a resident of New Mexico with a *Shigella flexneri* isolate genomically related (under 15 SNPs) to the outbreak strain. One patient had two separate positive samples collected less than 90 days apart, which was deemed not a separate incident case. Another patient had two samples collected > 90 days apart and was deemed two separate incident cases. Four samples were collected in NM, but upon interview or medical record review were out of catchment (OOC) and excluded from case data collection, for a total n = 202. Eighty-two (40.6%) cases were interviewed by New Mexico Department of Health (NMDOH) staff and the remaining cases underwent medical record review for variables including outcome, hospitalization, length of stay, symptoms, onset date, MSM activity, and housing status. Cases were defined as a Person Experiencing Homelessness (PEH) if either the interview or medical record reported homelessness at time of infection. We created a PEH-Adjacent category for cases who reported being unstably housed (e.g., living in a motel) or whose occupation brought them into direct contact with PEH (e.g., shelter worker, healthcare worker caring for an identified case). There was one case who was both MSM and PEH; based on epidemiologic and genomic evidence, this case was classified as PEH for the purpose of the study. We defined cases as “Sporadic” when they did not fit either PEH, PEH-Adjacent, Daycare, or MSM categories, but were still related to the outbreak.

### Sample collection and whole-genome sequencing

All *Shigella* cases are required by New Mexico Administrative Code to be reported to the New Mexico Department of Health, and associated specimens are sent to the State Scientific Laboratory Division (SLD), where confirmatory testing and whole-genome sequencing are performed as part of the Centers for Disease Control and Prevention (CDC)’s PulseNet program (Supplementary Data 2). Additionally, we sequenced 28 isolates via Oxford Nanopore GridION on a R9.4.1 flowcell using the Rapid Barcoding Kit 96 V14 (SQK-RBK114.96) to obtain long-reads and close the genomes. We chose one isolate that was the earliest viable NM isolate archived by NMDOH (PNUSAE076676, collected 06/22/2021) to be our internal reference strain for this outbreak.

### Genomic and phylogenetic analyses

Illumina short-read data was assembled and annotated using the *Bactopia* pipeline^12^. We performed hybrid assemblies using Nanopore long-reads and Illumina short-read data via Bactopia using Unicycler v.0.5.0^13^. This yielded a complete genome for our internal reference strain PNUSAE076676 (SRR15103314) which is accessioned under NCBI BioProject PRJNA1040311. AMRFinderPlus v.3.11^14^ was used to identify antimicrobial resistance genes.

We used the *snippy* v.4.6.0 (https://github.com/tseemann/snippy) pipeline with --*mincov 8* and otherwise default parameters to map short-reads against reference sequences, call variants, and to generate consensus genome alignments. We mapped all samples against two reference genomes, the standard external reference *Shigella flexneri* 2a str. 301 (NCBI accessions NC_004337 and NC_004851), and to our internal outbreak reference strain PNUSAE076676. Unless otherwise stated, all statistics are reported for the alignment to the external reference *Shigella flexneri* 2a str. 301.

We captured current transmission of closely related strains circulating in the U.S. by analyzing representative sequences from this outbreak in NM (n = 23) and 105 additional genomes that were less than 65 SNPs of the outbreak sequenced by CDC’s PulseNet program as part of the PDG000000004.4096 / PDS000007367.803 SNP cluster as defined by the NCBI Pathogen Database (https://www.ncbi.nlm.nih.gov/pathogens/) (Appendix 3). The resulting variable site alignment was 630 sites. Reference free alignments were performed using *ska2*^21^.

Maximum likelihood trees were calculated under the GTR + GAMMA model in IQ-Tree v.1.6.12^15^ with 10,000 ultrafast bootstraps and 10,000 bootstraps for the SH-like approximate likelihood ratio and visualized in ggtree^16^. BEAST v.1.10.4^17^ was used with a strict molecular clock to estimate time-resolved phylogenies for both internal and external reference alignments, and GrapeTree v.1.5.0^18^ to create minimum-spanning trees.

## Results

**Figure 1:**
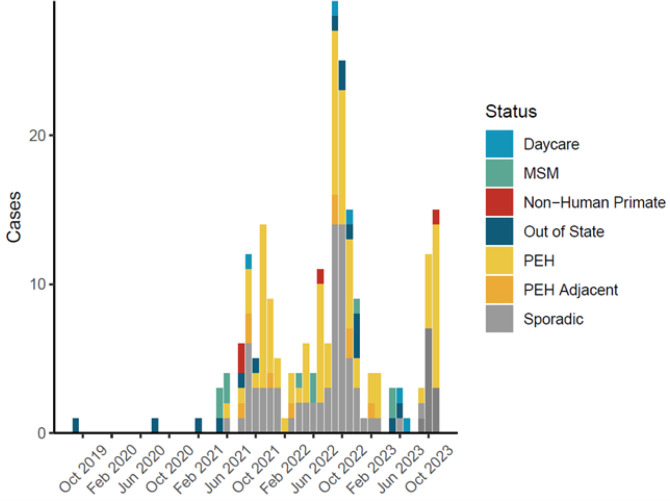
Epidemiological curve of a *S. flexneri* outbreak centered in Bernalillo County, New Mexico. Cases span from 2019 to 2023 and are colored according to status.

Between May 13, 2021, and November 19, 2023, NMDOH identified an outbreak of 202 *Shigella flexneri* serotype 2a cases in New Mexico (Fig. 1). NMDOH identified an initial cluster of four cases in May/June of 2021 among males reporting sexual activity with other men (MSM). However, by August 2021, cases spread into people experiencing homelessness (PEH).Over the course of the outbreak, half of the cases (n=102, 50.5%) were classified as either PEH or PEH-adjacent and eleven cases (5.5%) reported MSM activity (Fig. 1). All those who were not MSM or PEH, but still in the outbreak were deemed ‘sporadic’ (n=100, Fig. 1). Throughout the outbreak, the percentage of those who were PEH and sporadic remained roughly proportional. Nearly 70% of cases were hospitalized (n=141, 69.8%), with a median length of stay of four days (IQR 2.75–7.0 days). In contrast, only 40.8% of shigella cases not part of this outbreak in NM were hospitalized during the same period. Among PEH and PEH-adjacent cases, 78.6% (n=81) were hospitalized, compared to 60% (n=60) in sporadic and daycare cases. The high proportion of hospitalization in this outbreak may be due to differential healthcare-seeking behavior among PEH, leading to detection of only the most severe cases within the PEH population. The outbreak also spilled over into children attending daycare at several points (Fig. 1), but fortunately did not appear to cause prolonged transmission in daycare settings. Table 1 summarizes epidemiological and clinical characteristics of cases.

NMDOH epidemiologists collaborated with City of Albuquerque environmental health officials, local nonprofits, and other stakeholders, which resulted in installing portable toilets and handwashing stations near large encampments of PEHs. This was critical since many public restrooms had been closed due to the COVID-19 pandemic. An outreach point of contact (POC) with a local advocacy group was established to ensure services when PEHs were discharged from emergency rooms. Health Alert Network (HAN) messages were distributed to local providers informing them of significant developments in the outbreak.

**Figure 2:**
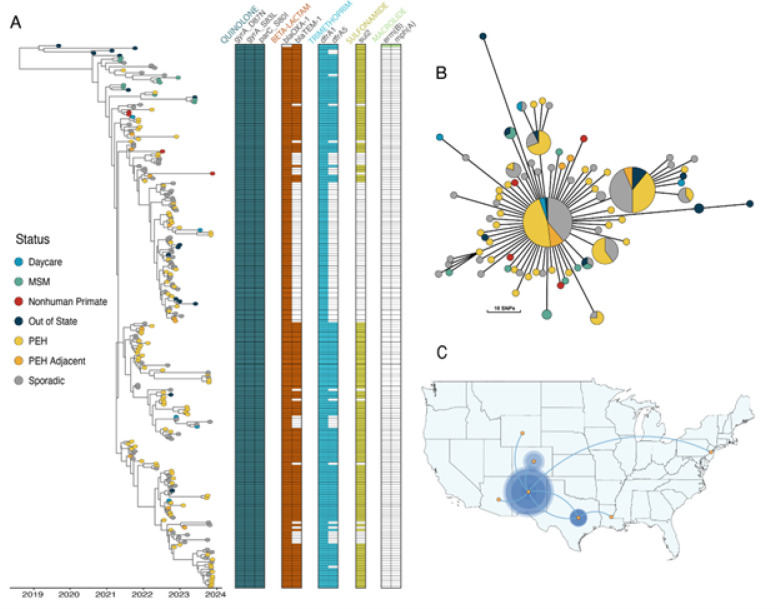
Outbreak strain phylogenies and transmission. **A.** Time-scaled BEAST phylogeny of outbreak strains. The outbreak clade includes 221 isolates, 202 residents of New Mexico, 15 out of state residents, and 4 nonhuman primates. Nodes are colored by demographic status. The presence of AMR genes are indicated and clustered by drug class. **B.** Minimum spanning tree of outbreak strains. The nodes are collapsed at 5 SNPs and the size of the circles are proportional to the number of strains within a given node. Nodes are colored by demographic status. Branch lengths correspond to the number of SNPs. **C.** Transmission inference from BEAST. The size of the circles denotes the number of cases and lines between locations indicate inferred transmission events.

**Figure 3:**
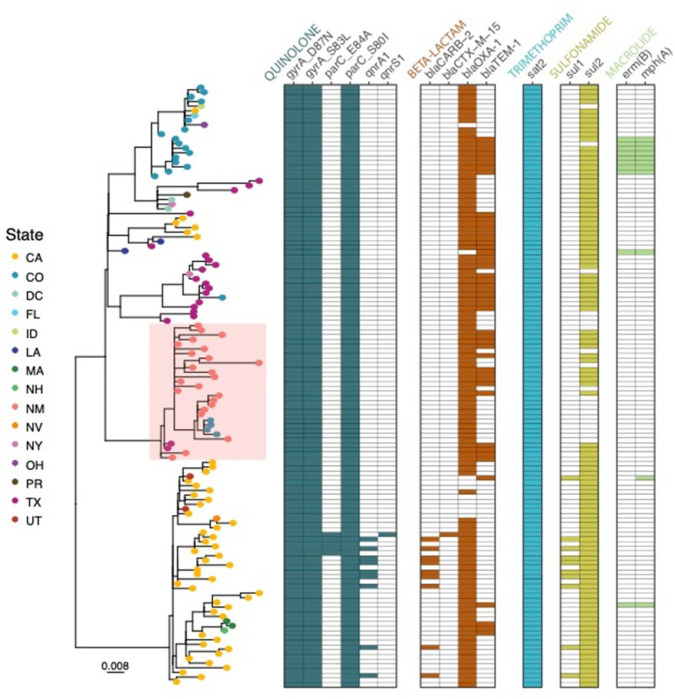
Maximum likelihood phylogeny of S. *flexneri2a* strains related to the New Mexico outbreak circulating within the U.S. We selected 23 representative sequences from the New Mexico outbreak (red box) and placed these in the context of 105 S. *flexneri 2a* strains that were less than 65 SNPs to the outbreak strain. Nodes are colored by U.S. state. The presence of AMR genes are indicated and clustered by drug class.

In order to determine if this outbreak strain was only localized to New Mexico, we pulled data from the NCBI Pathogen Detection database (https://www.ncbi.nlm.nih.gov/pathogens) and identified closely related strains to those found in NM. We determined our NM outbreak strains are part of the PDG000000004.4455/PDS000007367.963 SNP cluster defined by NCBI Pathogen Detection database and found 15 additional isolate genomes that were a part of the outbreak cluster but were not from NM. We pulled all sequencing data for a total of 220 samples assembled the genomes and also mapped the reads against the reference S. *flexneri* 2a str. 301 to identify single-nucleotide polymorphisms (SNPs). Our genomics analyses revealed that the outbreak isolates are highly clonal, with pairwise SNP differences ranging from 0–37 SNPs (IQR 8–17, median 12) (Fig 2B, Supp. Fig. 1). The outbreak strain harbors a set of triple point mutations (*parC_S80I, gyrA_D87N, gyrA_S83L*) conferring resistance to fluoroquinolones (ciprofloxacin). This strain also harbored a variety of other resistance genes (Fig. 2A), including the narrow spectrum beta-lactamase genes such as *blaOXA-1* and *blaTEM-1* which confer resistance to penicillins and first-generation cephalosporins, as well as *sul2* and *dfrA1* or *dfrA5*, conferring resistance to trimethoprim sulfamethoxazole. Despite the clonal nature of this outbreak, we noted that not all strains harbored the same resistance profile. In particular, a ~84Kb plasmid harboring a number of resistance genes, including the *blaTEM-1* gene, appears to be repeatedly lost across the outbreak (Fig. 2A). We did not detect macrolide resistance, *mph(A)* and *erm(b),* in isolates from NM (Fig. 2A, Fig. 3). Based on these analyses, NMDOH released a local Health Alert Network (HAN) notification on Feb 27, 2023 to all Albuquerque area providers alerting of this resistance pattern to ciprofloxacin.

To investigate the transmission and spread of this outbreak strain we created time-scaled phylogenetic trees. Our analyses indicate this strain was first detected in Louisiana in 2019 and Texas in 2020 before being introduced into NM. We estimated the date of the most recent common ancestor (MRCA) for all NM strains to August of 2020 [95% HDP 04/2020 – 12/2020], suggesting this strain was circulating in NM prior to the first recognized case in May 2021. While the first cases in NM reported MSM activity, these cases were not directly linked to the other nine MSM cases later in the outbreak, which are largely interspersed with the other cases (Fig. 2). One notable exception is a well-supported clade (posterior probability, PP = 1) linking two MSM cases from NM in April/May 2022 to two MSM cases from NM and one case in New Jersey (collected within days of each other) in May 2023 (Fig. 2). The strain quickly moved into circulating predominantly within the PEH community starting in June 2021. While we do find instances of clusters of PEH cases, these isolates are also interspersed across the phylogeny. The sporadic cases are also interspersed throughout the phylogenetic tree, indicating a complex transmission network across different populations. In addition, there were five daycare cases, again distributed throughout the phylogeny. This strain has spread from NM to include six Colorado cases, two from Arizona, and single cases from New Jersey and Wyoming (Fig. 2, Supp. Fig. 5), but does not appear to have caused similar outbreaks in these locations. We also contextualized this outbreak within the broader S. *flexneri* 2a diversity (Fig. 3). We find that our outbreak forms a distinct cluster, but sits within other closely related lineages that have been circulating in the U.S. (Fig 3). Concerningly, many of these closely related strains harbor resistance genes towards macrolides or third-generation cephalosporins, which if transferred into our outbreak strain would confer an XDR phenotype. Overall, our phylogenetic analyses point to a complex transmission network that spans MSM, PEH, daycare populations, and other U.S. states.

In an effort to increase the resolution in our analyses, we sequenced the earliest viable NM isolate archived by NMDOH (PNUSAE076676, collected 06/22/2021) via Oxford Nanopore long-read technologies. This isolate was the 6th isolate collected in the NM outbreak and temporally, 40 days from the first identified case on May 14, 2021. Combining the long and short-reads in a hybrid assembly yielded a fully closed genome, which we designated our internal outbreak reference strain. We mapped all isolates against the closed PNUSAE076676 genome and created phylogenies from the resulting alignments. Due to the clonal nature of the outbreak, some nodes in the phylogenies have low support and we have taken a conservative approach in making inferences based on these phylogenies (Supp. Figs. 2,3,4,5). This limitation is likely to be a reality for many bacterial outbreaks where the rate of mutation accumulation is much slower than the transmission rate during the outbreak^19^.

Remarkably in August of 2021, *Shigella* was detected within the Albuquerque Biopark, in seven out of eight Western lowland gorillas (*Gorilla gorilla*), four out of four siamangs (*Hylobates syndactylus*), three out of four Sumatran orangutans (*Pongo abelii*), and two out of nine chimpanzees (*Pan troglodytes*). The veterinary clinical management of this event is described in Bradford et al.^20^ One gorilla, aged 48, and three siamangs, including a 32-year-old male, a 30-year-old female, and her two-month-old infant, died as a result of the *Shigella* infection. BioPark staff declared quarantine after the first animals became symptomatic and worked closely with NMDOH to implement biosafety protocols, including disinfection, personal protective equipment (PPE) use, and cohorting of staff to prevent further transmission. NMDOH, in collaboration with City of Albuquerque Environmental Health Department and the BioPark Zoo, re-interviewed available cases using a specialized shigellosis questionnaire focusing on areas around the zoo, water exposure from the Rio Grande flood drainage area, and produce-specific questions based on invoices from the zoo’s dietary ordering. Despite extensive investigation, the NMDOH team found no significant epidemiological connection. No BioPark zoo staff reported illness compatible with shigellosis. In July 2022, almost a year after the initial outbreak, a 22-year-old chimpanzee began exhibiting symptoms, tested positive for S. *flexneri* via BioFire enteric pathogen panel, and subsequently died. No other primate illnesses were reported at that time.

The remaining siamang of the troop (named “Eerie”) was transferred to an out of state zoological park (Zoo B) in 2021, after four negative cultures and a negative PCR test. Unfortunately, after Eerie’s introduction, a *Shigella* outbreak occurred in Zoo B, which killed an additional two siamangs. Eerie remained asymptomatic, and was treated at Zoo B with amoxicillin-clavulanic acid (a penicillin) and ciprofloxacin (a fluoroquinolone) to eradicate carriage. No cultures were taken during the Zoo B outbreak. Eerie was then transferred back to the Albuquerque BioPark Zoo, where he tested positive for *Shigella*. Unfortunately, despite treatment with appropriate antibiotics and an attempted transfaunation, he was euthanized in February 2024.

We obtained genome sequences of bacterial isolates from four infected NHP (a gorilla and an orangutan in 2021, chimpanzee in 2022, and Eerie’s November 2023 isolate). Our analysis places the NHP strains directly within, and connected to, the human outbreak circulating in the Albuquerque metro area. (Fig. 2). These results demonstrate that the outbreak strain was circulating in the human population and was subsequently introduced into the NHP population within the BioPark. This analysis also suggests that Eerie was a long-term asymptomatic carrier of this strain. When comparing pairwise SNPs, we find that all NHP samples have their closest pair with a human sample, ranging from 2 – 13 SNPs. For instance, we found that both NHP samples from August 2021 are only 2 SNPs away from the same Sporadic human sample from October 2021. Pairwise SNP distance between just the NHP samples ranged from 3–17 SNPs. As expected, the strains sampled from NHPs were multidrug resistant and shared the resistance profiles with the human strain. All NHP strains were resistant to fluoroquinolones due to the presence of the triple point mutations mentioned above and harbored the *blaOXA-1* conferring resistance to penicillins. All strains, except for the 2023 siamang strain, also harbored *blaTEM-1*.

A major question was whether the 2022 chimpanzee infection was due to asymptomatic carriage by either a zookeeper or another animal, or was the result of another separate introduction into the zoo. While it is likely that Eerie, the siamang, was a long-term carrier, this animal was not present at the time of the 2022 infection. The July 2022 chimpanzee sample is 17 SNPs different from the 2023 siamang sample, while only 5 SNPs away from two human samples, collected in late 2021 and in March 2022. The 2023 siamang was 5 SNPs different from 3 human samples collected in September 2021, November 2021 and January 2022. Due to the highly clonal population structure and resulting small number of SNPs, definitively answering this question is not trivial. When mapping to the external reference, our phylogenetic reconstruction does not place the 2022 chimpanzee isolate with any of the other NHP isolates (Fig. 2, Supp. Fig. 2,3). However, when mapping to our internal outbreak reference our phylogenies show the chimp 2022 isolate groups as a descendant of one of the 2021 NHP samples, albeit with low support (Supp. Fig. 3). In an attempt to gain more resolution, we performed additional long-read Nanopore sequencing on 28 isolates that spanned across the outbreak, but crucially included all four of the nonhuman primate samples. The long-read data was combined with short-read data to yield dramatically improved assemblies and resulted in complete or near-complete genomes for all 28 samples. To avoid potential reference bias, we utilized *ska2*^21^, which is a k-mer based, reference free alignment tool. However, even with this dataset, the placement of the NHP samples are largely unresolved, essentially forming various branches stemming from a single “trunk” as one large polytomy in the phylogeny. However, the fact that in no phylogenetic analyses do we see all NHP samples grouping together does lend weight to the possibility of multiple introductions into the zoo.

## Discussion

We have described a multi-state, multi-species, multidrug-resistant outbreak of *S*. *flexneri* 2a involving different human populations and remarkably, nonhuman primates. To our knowledge, this is the first time it has been definitively shown that the same *Shigella* outbreak strain affecting humans crossed species and caused infections in primate populations. This was one of the largest identified outbreaks of shigellosis in New Mexico, and the first large outbreak involving a multidrug-resistant strain. While we have not detected macrolide or extended-spectrum beta-lactam (ESBL) resistance in this strain, the fact that many *Shigella* strains circulating in the U.S., including in NM, harbor these resistance genes warrants vigilant surveillance.

This outbreak impacted various groups, such as MSM, PEH, daycare attendees, and the general public. Due to the significant impact and severity of this outbreak—which accounted for 39.3% of all shigellosis cases in New Mexico during the period and led to a 69.8% hospitalization rate—the New Mexico Department of Health (NMDOH) implemented aggressive measures, including establishing partnerships to ensure patient care for vulnerable groups, conducting thorough epidemiological investigations, and issuing local health alerts to inform clinical providers about the outbreak and the associated resistance patterns of the strain. It is likely that the outbreak was much larger than reported cases, since not every *Shigella* case in NM has an associated isolate, which we used to define this outbreak. Additionally, care-seeking behavior among affected populations may vary. For example, the high hospitalization rate in PEH may stem from varied healthcare-seeking behavior, resulting in the detection of only severe cases.

The use of active public health surveillance through the FoodNet programs provided valuable data in a population notoriously difficult to interview^4^. Collaborating with local nonprofits and community partners allowed for the development of trust and ability to provide care to vulnerable populations. Our results spurred direct public health action as our data alerted local clinicians and veterinarians to not treat shigellosis cases with fluoroquinolones, but rather to use macrolide antibiotics. Without the genomics data, it is likely that both the connectedness of these cases and the AMR patterns would have gone unrecognized.

This outbreak was particularly devastating in the NHP population within the Albuquerque BioPark Zoo. The BioPark participates in the Association of Zoos and Aquariums Species Survival Plan (SSP), which ensures genetic diversity among captive populations^22^. The loss of these endangered animals, particularly those of reproductive age, is a setback to achieving the goals of the SSP. Despite aggressive investigation by the Albuquerque BioPark and NMDOH, it is unclear how this *S. flexneri* outbreak strain was introduced into the zoo environment, which our phylogenies indicate may have occurred on multiple occasions. Of note, no zoo staff reported illness consistent with shigellosis during this time period. As *Shigella* is known to persist on surfaces^23^, it may have been introduced when a zoo visitor threw a contaminated item into the enclosure. It may also have been introduced on cardboard tubes used as enrichment items, although this common zoo practice was discontinued as part of the initial response to the 2021 primate infections, and cannot explain the 2022 chimpanzee infection. Another possibility is that *Shigella* may have been introduced on an insect species acting as a mechanical vector. Houseflies (*Musca domestica*) are able to carry infectious doses of *Shigella* species and have been associated with *Shigella* infections ^24,25^, suggesting a housefly or other insect vector could introduce the bacteria into the zoo. Insect species acting as mechanical vectors could explain some of the genomic diversity among the NHP samples.

Our report, as well as others ^26–28^, demonstrates that *Shigella* infections in primate populations can have severe impacts. A recent study detected various pathogenic *E. coli and Shigella* within wild gorillas and chimpanzees in Cameroon and Tanzania^29^. Thus, there is potential real risk to wild primate populations, as contact between humans and these animals increases - particularly in areas of high human shigellosis prevalence. The rising rates of AMR within *Shigella*, therefore, have direct implications for human and primate health in the future.

## Figures and Tables

**Table 1:** Characteristics Among New Mexico *Shigella* Cases, May 2021 – July 2023

Variables	Total (%) N = 202
**Gender**	
Female	63 (31.2)
Male	139 (68.8)
**Race**	
Asian	1 (0.5)
Black	9 (4.5)
American Indian/Alaskan Native	33 (16.3)
Multi-Racial	11 (5.4)
Other	11 (5.4)
White	127 (62.8)
Unknown	10 (4.9)
**Ethnicity**	
Hispanic	89 (44.1)
Non-Hispanic	103 (50.7)
Unknown	10 (4.9)
**Age (years)**	
0–4	11 (5.4)
5–17	11 (5.4)
18–45	100 (49.5)
46–64	64 (31.7)
65+	16 (7.9)
**Experiencing Homelessness**	
Yes	93 (46.1)
PEH Adjacent	10 (4.9)
No/Unknown	100 (49.5)
**Men Who Have Sex With Men (MSM)**	
Yes	10 (4.9)
No/Unknown	191 (94.5)
**HIV Status**	
Positive	19 (9.4)
Negative/Unknown	183 (90.6)
**Illicit Substance Use**	
Yes	56 (27.7)
No/Unknown	146 (72.3)
**Fever**	
Yes	124 (61.4)
No/Unknown	78 (38.6)
**Diarrhea**	
Yes	199 (98.5)
No/Unknown	3 (1.5)
**Bloody Diarrhea**	
Yes	122 (60.4)
No/Unknown	80 (39.6)
**Hospitalized**	
Yes	141 (69.8)
No/Unknown	61 (30.2)

## Data Availability

Sequencing data from this project has been deposited under NCBI Bioproject PRJNA1040311. All short-read data can be accessed in the NCBI Sequence Read Archive using the accessions listed in the supplementary material (Supplementary data 2 & 3). Alignments, results files and full size figures are available at https://github.com/DommanLab/shigella-flex-NM.
